# Pathophysiology of spontaneous coronary artery dissection: hematoma, not thrombus

**DOI:** 10.3389/fcvm.2023.1260478

**Published:** 2023-10-20

**Authors:** Aleksandra Djokovic, Gordana Krljanac, Predrag Matic, Rastko Zivic, Vuk Djulejic, Marija Marjanovic Haljilji, Dusan Popovic, Branka Filipovic, Svetlana Apostolovic

**Affiliations:** ^1^ Faculty of Medicine, University of Belgrade, Belgrade, Serbia; ^2^ Department of Cardiology, University Hospital Center Bezanijska Kosa, Belgrade, Serbia; ^3^ Cardiology Clinic, University Clinical Center of Serbia, Belgrade, Serbia; ^4^ Clinic for Vascular Surgery, Institute for Cardiovascular Diseases “Dedinje”, Belgrade, Serbia; ^5^ Department for Surgery, Clinical Hospital Center Dr Dragisa Misovic “Dedinje”, Belgrade Serbia; ^6^ Faculty of Medicine, Institute of Anatomy, Belgrade, Serbia; ^7^ Department for Gastroenterology, Clinical Hospital Center Dr Dragisa Misovic “Dedinje”, Belgrade Serbia; ^8^ Coronary Care Unit, Cardiology Clinic, University Clinical Center of Nis, Nis, Serbia; ^9^ Faculty of Medicine, University of Nis, Nis, Serbia

**Keywords:** spontaneous coronary artery dissection, acute coronary syndrome, pathophysiology, women’s health, pregnancy, fibromuscular dysplasia

## Abstract

Spontaneous coronary artery dissection (SCAD) accounts for 1.7%–4% of all acute coronary syndrome presentations, particularly among young women with an emerging awareness of its importance. The demarcation of acute SCAD from coronary atherothrombosis and the proper therapeutic approach still represents a major clinical challenge. Certain arteriopathies and triggers are related to SCAD, with high variability in their prevalence, and often, the cause remains unknown. The objective of this review is to provide contemporary knowledge of the pathophysiology of SCAD and possible therapeutic solutions.

## 1. Introduction

Spontaneous coronary artery dissection (SCAD) is an often-underrecognized clinical condition primarily associated with acute coronary syndrome (ACS) in young or middle-aged women, which can have fatal consequences. The utilization of intracoronary imaging techniques and the introduction of an angiographic classification by Saw J et al. in 2014 (1), as well as SCAD position papers published by the European Society of Cardiology and American Heart Association in 2018 (2, 3), have contributed to the increased recognition and prevalence of SCAD. In the USA, the estimated prevalence of SCAD in ACS ranges from 1.7% to 4% (4), while it varies from 3.1% to 9.7% in patients with premature myocardial infarction (less than 45 years) (5–7), and up to 43% in females experiencing ACS during the peripartum period (8). A meta-analysis of 2,172 SCAD patients conducted by Franke KB et al. reported that 84% of the cases involved females, with a mean age of 51 years, and significant heterogeneity across studies regarding baseline characteristics and outcomes analyzed (9). While the majority of patients typically present with characteristic chest pain (96%) and show elevated cardiac biomarkers (10), a small subset (0.4%–4%) may exhibit normal cardiac troponin levels (11). Additionally, SCAD has been identified as a potential cause of sudden cardiac death (SCD) in 3%–11% of cases, which raises the possibility of an underestimation of SCAD prevalence. This observation highlights the challenge in accurately assessing SCAD's true frequency, particularly given the limited data available from postmortem cases (4). Timely diagnosis of SCAD is crucial due to its distinct pathophysiology and management compared to atherosclerotic disease.

## 2. Anatomy and physiology of coronary arteries

Coronary arteries arise from the aortic root, which is the initial segment of the ascending aorta. The right coronary artery (RCA) originates from the right sinus of Valsalva and enters the atrioventricular groove, descending anteriorly and inferiorly along the right border of the heart, giving rise to several branches before passing posteriorly and inferiorly (12). The left coronary artery, the main stem, originates from the left sinus of Valsalva, travels anteriorly and to the left between the left atrial appendage and the pulmonary trunk, dividing shortly into the circumflex (Cx) and anterior interventricular (or descending) (LAD) arteries (13, 14). Although coronary vessels were traditionally considered branches of the aorta, recent studies on mouse models have shown that coronary arteries derive from cells originating from the sinus venosus, the venous inflow tract of the primitive heart. These cells migrate to the muscle layer and form a vascular plexus that subsequently remodels into arteries (15). The vascular supply of arteries larger than 0.5 mm is provided by vasa vasorum, which traverse the adventitia but not the muscular layer of the vessel wall due to luminal compressive forces. Vasa vasorum can be classified into vasa vasorum interna, originating from the luminal surface or media and penetrating the vessel wall toward the adventitia, and vasa vasorum externa, primarily located in the adventitia and originating from various anatomical points (16).

The adventitia represents a vital component of the vessel wall, regulating the inflammatory response and contributing to vessel repair. However, its impaired ability to respond adequately to injury is implicated in both spontaneous dissection and atherosclerotic processes (17). In a study analyzing the mechanical properties of coronary arteries *in vitro*, Claes E et al. investigated five human donors. They found that the responsiveness of coronary arteries to wall stress follows a typical J-curve pattern, with the initial portion dependent on elastin and the stiffer segment reliant on collagen. This relationship may shift upward over time due to aging or distinct pathologies. While coronary arteries are considered elastic, their circumferential strength is significantly lower than that of the aorta and declines progressively with age, with a more pronounced decrease occurring around the ages of 30–40 years (18).

The pressure within the internal vasa vasorum is lower than that in the coronary artery, supported by the firmness and elasticity of the endothelium. However, when the endothelium becomes infiltrated with inflammatory cells and cytokines, it becomes compromised, leading to vessel wall disruption and the transmission of pressure from the coronary artery lumen to the subintimal layer (16).

## 3. Definition and classification of SCAD

The first autopsy report on SCAD was published in 1931 (19). Since then, there has been increasing recognition of SCAD, leading to numerous publications in the past decade and highlighting the need for a precise definition of this condition. SCAD is currently defined as the spontaneous tearing of the coronary artery wall, resulting in the formation of an intimal hematoma that obstructs blood flow. It is important to note that the term SCAD specifically excludes iatrogenic dissections (those induced by medical procedures) and those associated with trauma. Additionally, the contemporary definition of SCAD excludes cases involving atherosclerotic coronary artery disease.

**Figure 1:**
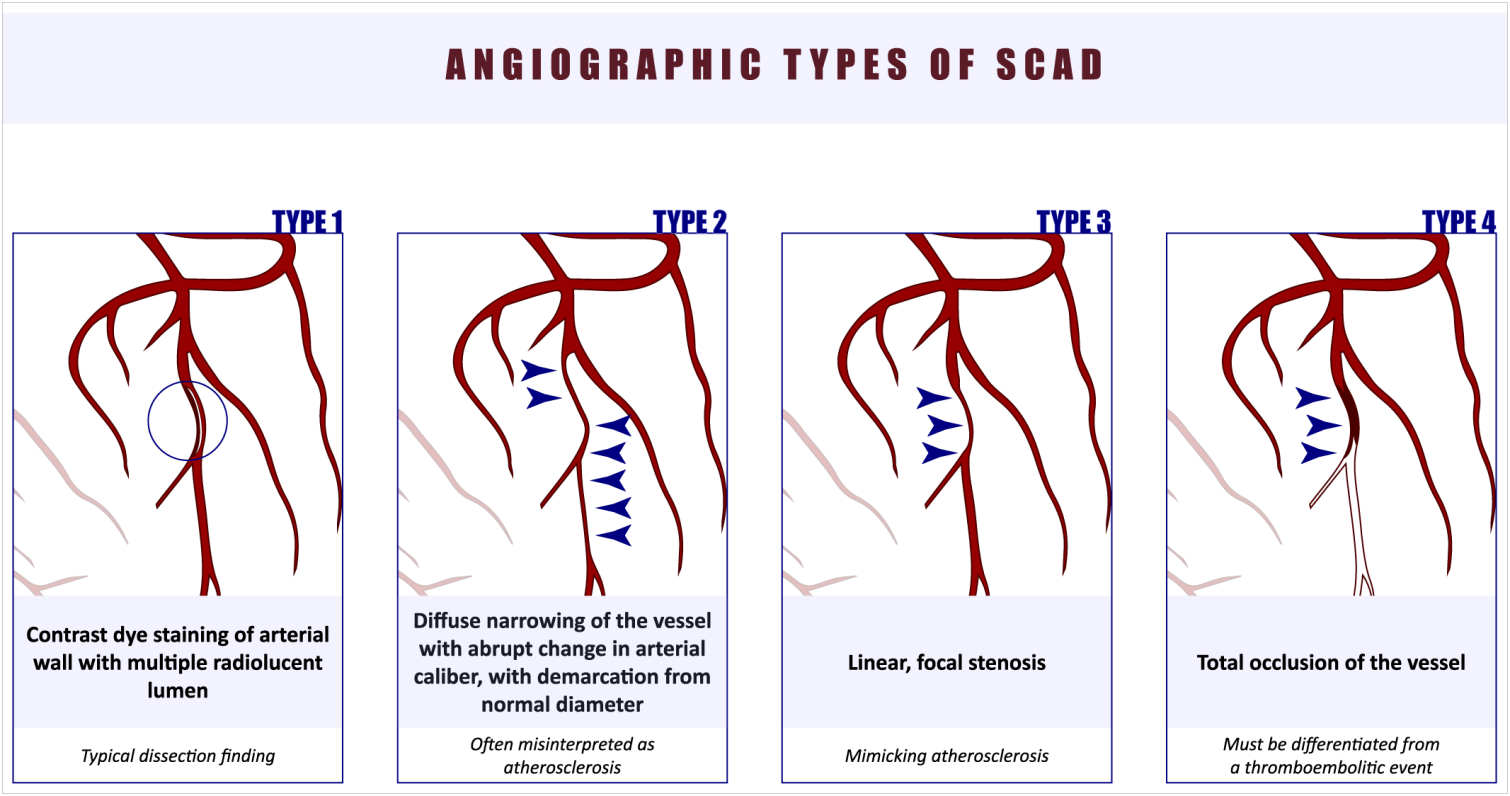
Four different angiographic types of SCAD.

Initially, SCAD was classified into four angiographic types (1) (Figure 1). Type 1 is considered pathognomonic, characterized by contrast staining of the coronary artery wall and the presence of a false lumen. Type 2 SCAD is often misdiagnosed as it presents as a diffusely narrowed segment, commonly involving the medial or distal parts of the artery, with a subtle demarcation line separating it from the true caliber of the coronary artery. Type 2 SCAD is further divided into Type 2A, where normal arterial segments can be observed proximal and distal to the SCAD segment, and Type 2B, where the dissection extends to the distal tip of the artery (20). Type 3 SCAD is challenging to diagnose due to its focal appearance. More recently, Type 4 SCAD was added to the classification, characterized by total occlusion of the vessel, which must be differentiated from a thromboembolic event (21). Tanis et al. proposed an alternative classification based on the etiology of SCAD, dividing it into four subtypes: peripartum, atherosclerotic, idiopathic, and those related to connective tissue disorders (22).

## 4. Proposed mechanisms of SCAD

**Figure 2:**
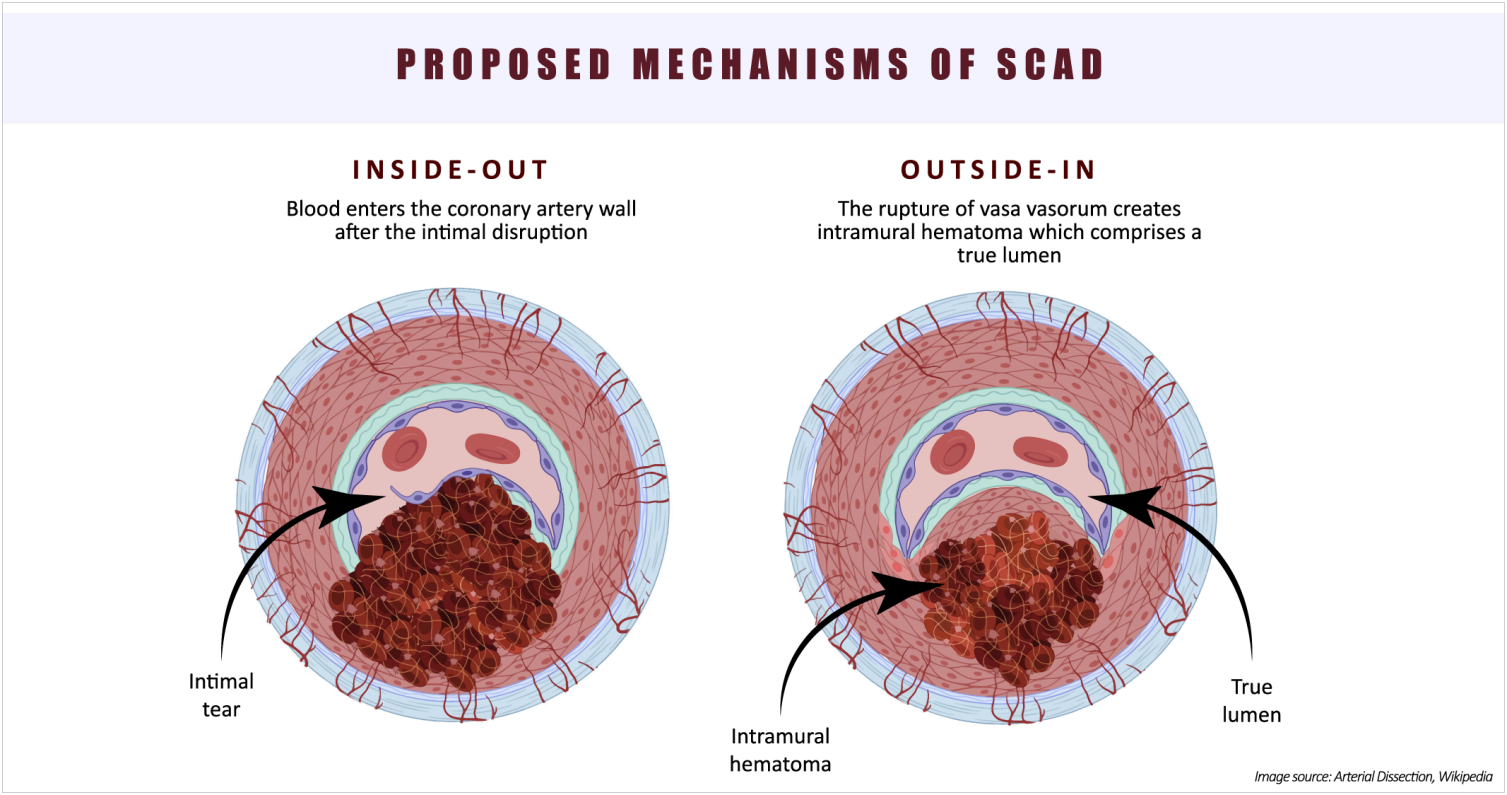
Proposed mechanisms of SCAD occurrence.

The etiology and pathophysiology of SCAD still present many unresolved questions. The existence of two types of SCAD—one with an intimal tear and blood entering the vessel wall (“inside-out”), and the other without an observed intimal tear, hypothesized as a consequence of vasa vasorum rupture (“outside-in”)—complicates our understanding of the underlying cause of this condition (20) (Figure 2). In an observational study of 65 SCAD patients, optical coherence tomography (OCT) was utilized to investigate false lumen formation (23). The absence of fenestration in the false lumen results in increased pressure, leading to compression of the true lumen. The authors proposed that the “outside-in” theory may unify the pathophysiology of SCAD. However, the lack of studies supporting the hypothesis of intramural bleeding with subsequent false lumen formation keeps the “inside-out” theory alive. Furthermore, the theory that intimal hemorrhage generates enough pressure to compromise flow in the true lumen requires further investigation through mechanical stress studies (24). An OCT study by Kwon et al. reported a higher density of adventitial vasa vasorum in SCAD patients compared to those with non-obstructive atherosclerotic coronary artery disease (CAD), suggesting that this anatomical feature may contribute to the occurrence of SCAD (25).

The role of microvasculature in the pathophysiology of SCAD remains unclear. It is reasonable to assume that microvascular dysfunction, which has been implicated in various cardiovascular diseases, plays a role in the pathophysiology of SCAD as well. However, specific studies addressing this aspect are currently lacking.

Inflammation and abnormalities of connective tissue have been proposed as important factors in the pathophysiology of SCAD (26). Autopsy reports frequently describe infiltration of the adventitia surrounding SCAD with inflammatory cells, although the true relationship has not been proven or well understood (27). Based on autopsy study findings, segmental eosinophilic infiltration has been identified as a pathognomonic feature of non-atherosclerotic SCAD (28). A small retrospective study by Canga et al. reported significantly higher levels of systemic inflammation markers (white blood cell count, neutrophil-to-lymphocyte ratio, C-reactive protein) in SCAD patients compared to controls, similar to patients with CAD, but the causal relationship between inflammation and SCAD remains uncertain (29). The perivascular adipose tissue attenuation around coronary arteries, as determined by coronary computed tomography angiography (CCTA), represents a novel imaging marker for assessing inflammation burden. In a recent study, it was discovered that 48 SCAD patients exhibited higher pericoronary adipose tissue attenuation when compared to patients without SCAD, indicating an elevated perivascular inflammatory activity within this patient population (30).

A study by Margarits M et al. compared the coronary and myocardial histology and immunohistochemistry findings of 36 autopsy SCAD cases with 359 SCAD survivors (31). Autopsy cases were predominantly characterized by single-vessel disease, with a higher proportion involving the left main artery. Two-thirds of autopsy cases did not develop myocardial infarction. Inflammation was more pronounced in the dissected region, suggested to be a reaction rather than the cause of SCAD, and was more intensively present in SCAD survivors. Approximately 47% of autopsy cases exhibited limited intimal fibro-elastic thickening without features of fibromuscular dysplasia (FMD) or abnormalities in the endothelial or internal elastic lamina. There were no differences in vasa vasorum density between SCAD cases and controls.

The genetic predisposition for SCAD is not yet fully understood. Genetic studies indicate that a small percentage of SCAD patients carry distinct gene mutations (32). Additionally, SCAD occurs frequently in patients with FMD and migraine, and recent genetic studies have identified shared risk variants between these conditions and SCAD (33). In a meta-analysis comprising 1,917 cases and 9,292 controls, Adlam et al. identified 16 risk loci for SCAD, primarily associated with genes involved in vascular muscle cells and fibroblasts (34). Based on a specific risk locus for factors initiating coagulation, the authors concluded that arterial integrity, along with tissue-mediated coagulation, should be considered in the pathophysiology of SCAD, opening up new possibilities for management and prevention. The shared genetics found between hypertension and SCAD also suggest the need for future studies on possible similar biological pathways in SCAD and CAD.

### 4.1. Hormonal influences and SCAD

Appreciating the fact that the typical SCAD patient is a woman aged between 44 and 53 years, it is reasonable to assume that sex hormones play an important role in SCAD pathophysiology.

Data from the SWED-PREG registry, which includes women diagnosed with SCAD between 1997 and 2019 and under 50 years of age, revealed that 28% of cases were associated with pregnancy (during pregnancy or within 14 weeks postpartum) (35). Significant changes occurring during this period are thought to contribute to the pathophysiology of SCAD. Hemodynamic, respiratory, and metabolic changes lead to a substantial increase in blood volume, placing high demands on the cardiovascular system and causing cardiomyocytes to change their elasticity and contractility (36). Additionally, vascular overload during pregnancy can induce changes in the endothelium, increasing its permeability and reducing coagulability (37). These changes occur early in pregnancy, as the placenta begins producing various molecular signals such as hormones and growth factors, which affect the peripheral vasculature (38). Hormonal changes during pregnancy weaken the media layer of the vessel wall due to decreased collagen production and alterations in collagen metabolism. In the past, it was speculated that higher levels of progesterone cause the loss of elastic fibers, making blood vessels during pregnancy more susceptible to dissection, particularly in response to high cardiac output and smooth muscle cell hypertrophy (39). However, these generalized vascular changes have not been conclusively proven in postmortem studies (40). During labor and delivery, cardiac output increases, and shear stress on the vessels rises significantly, further increasing the risk of SCAD development (41). As a result, SCAD is most commonly observed in the third trimester or postpartum period among causes of acute myocardial infarction (AMI) during pregnancy (42).

The potential for SCAD recurrence during subsequent pregnancies, often with serious consequences, necessitates clear guidance for peripartum SCAD survivors. In a cohort study involving 54 females who experienced pregnancy-associated SCAD, 15% encountered a recurrent SCAD event within a 5-year follow-up period, with 50% of these recurrences happening within 3 months after delivery (43). More recently, within a cohort of 636 women of childbearing age, 23 chose to become pregnant following a prior SCAD episode. Remarkably, most of these women managed pregnancy and lactation without any evidence of an increased risk of SCAD recurrence compared to women with a history of SCAD who did not become pregnant (44). Furthermore, in a meta-analysis involving 4,206 SCAD patients, pregnancy did not exhibit a significant association with recurrent SCAD (45).

While it might initially appear reasonable to advise peripartum SCAD survivors to avoid future pregnancies due to the unpredictable nature of SCAD (46), this recommendation is not aligned with the current consensus among experts (3). This is primarily because the majority of these patients proceed to have uncomplicated pregnancies. Instead, the recommended approach is to offer comprehensive pre-conception counseling, encouraging close collaboration between gynecologists, obstetricians, and cardiologists.

Data regarding the association between hormone replacement therapy (HRT) and oral contraceptives with SCAD are scarce. In a case report from 2011, Zehir et al. described a 36-year-old woman with a history of 7 years of third-generation oral contraceptive use who presented with AMI due to left anterior descending artery dissection and proximal thrombotic occlusion of the right coronary artery (47). Changes in the architecture of the coronary artery wall caused by estrogen have been previously discussed as a possible explanation for the increased risk of SCAD. Oral contraceptives could potentially contribute to SCAD risk through their effects on coagulability, vasomotor control, and oxidative stress. However, in a prospective study of non-atherosclerotic SCAD patients by Saw et al., the prevalence of oral hormonal therapy use was 10.7%, without a significant causative effect (48). In the aforementioned study by Tweet et al., there was no significant difference in the prevalence of previous hormonal therapy between pregnancy-related and non-pregnancy-related SCAD patients, with rates of administration being 28% and 16%, respectively (43).

Two cases of SCAD in young females were reported in 2003, both occurring during the menstrual period (49). The authors speculated that the drop in estrogen and progesterone levels, similar to the peripartum period, triggers the loss of hormonal vascular smooth muscle cell suppression and increases smooth muscle activity, leading to coronary media frailty.

If we accept that estrogen and progesterone contribute to vessel wall weakness and predispose individuals to SCAD, it would be reasonable to assume that menopausal women without HRT are at lower risk for SCAD. In a study comparing clinical features, angiographic findings, management, and in-hospital outcomes of 245 women with SCAD based on their menopausal status, it was found that premenopausal women with SCAD had a higher clinical and angiographic risk profile, including a higher prevalence of more proximal localization and larger infarct size, leading to a higher prevalence of left ventricular systolic dysfunction (50). In contrast, postmenopausal women had a higher prevalence of standard atherosclerotic risk factors. However, there were no significant differences in terms of in-hospital major adverse cardiovascular events (MACE) between the groups.

Finally, there is limited data on a possible association between hypothyroidism and SCAD. Observations that individuals with hypothyroidism tend to have a higher prevalence of spontaneous dissections, primarily in the aortic, carotid, and vertebral arteries, have led to speculation that it may be related to SCAD as well. Patients with hypothyroidism are also more susceptible to iatrogenic SCAD. In a recent observational multicenter study of 73 SCAD patients, a significantly higher rate of hypothyroidism was observed compared to matched controls with atherosclerotic acute coronary syndrome and no evidence of coronary artery dissection (51). Possible pathways for the development of SCAD in hypothyroidism involve interstitial fluid retention and increased production of hyaluronic acid by fibroblasts, leading to the repression of smooth muscle cells and resulting in a weaker vessel wall. However, these speculations require further study and clearer demonstration in future research.

### 4.2. Connective tissue disorders and SCAD

Genetic studies of SCAD survivors have reported the identification of mutations associated with Marfan syndrome and Ehlers-Danlos syndrome in previously undiagnosed cases (52). In a large prospective multicenter study of 750 SCAD patients, the prevalence of connective tissue disorders (CTDs) was found to be 3.6%, and they were identified as independent predictors of 30-day major adverse cardiovascular events (27). CTDs, such as Ehlers-Danlos syndrome, Marfan syndrome (MFS), Autosomal Dominant Polycystic Kidney Disease, Loeys-Dietz syndrome, and Pseudoxanthoma elasticum, are rare hereditary conditions, and SCAD might be the initial presentation of an underlying connective tissue disorder (53). These disorders share a common feature of inadequate structure and synthesis of the extracellular matrix, as well as alterations in elastic fibers, contributing to their multisystemic nature. In fact, there are thirty-six different clinical entities, involving more than 40 different genes or gene loci, that should be differentiated from the most well-known condition, Marfan syndrome (MFS) (54).

Genetic studies, as well as cellular and molecular investigations of SCAD, have revealed disruptions in tumor growth factor beta (TGF-β) signaling, changes in the extracellular matrix, cytoskeleton, and metabolism caused by specific gene mutations, including LRP1, collagen genes, fibrillin, and TGF-β receptors. These genetic mutations are shared between many connective tissue disorders and SCAD (55).

Given that SCAD may be the initial manifestation of an underlying connective tissue disorder, it is important to include genetic testing for CTDs in the diagnostic algorithm for SCAD. Furthermore, it can be speculated that all SCAD patients are actually individuals with unrecognized connective tissue disorders, with genetic mutations expressed in female patients, who develop dissections in the presence of precipitating factors.

### 4.3. Fibromuscular dysplasia and SCAD

In addition to being a typical event in females, SCAD is often considered a severe manifestation of underlying systemic arteriopathy, with FMD emerging as a synonymous condition for SCAD.

FMD, a non-inflammatory and non-atherosclerotic disease of unknown etiology, was first described in 1958 by McCormack et al. in patients with renovascular hypertension (56). Initially considered hyperplasia, further studies reclassified it as dysplasia. Typical angiographic findings of FMD include two types: multifocal stenoses with “string of beads” appearance and focal lesions, primarily observed in renal and cranial vessels. Pathological classification of FMD into intimal, medial, and perimedial disease is based on the location of irregularly arranged mesenchymal cells within a loose matrix of subendothelial connective tissue and a fragmented internal elastic lamina (57). In modern practice, the diagnosis of FMD is primarily achieved through imaging techniques, rendering the pathological classification obsolete in routine clinical use. It's important to highlight that, in addition to dissection, typical clinical FMD phenotypes encompass aneurysms and marked tortuosity in the affected arteries.

One of the early studies on FMD of coronary arteries was a case series by Pate et al. in 2005, reporting seven patients with previously documented renal FMD and typical angiographic findings of diffuse obliterative changes in middle or distal segments, predominantly in the LAD, with a clear demarcation line from the apparently healthy proximal segment (58). Prior to this study, coronary FMD was primarily diagnosed through autopsies, making this report unique in its assumption of specific angiographic findings for a condition that was considered a histopathological diagnosis. Another case series by Saw et al. in 2012 was the first to study SCAD in coronary FMD, reporting six females presenting with ACS (59). In the same year, a retrospective single-center cohort study of 87 SCAD patients reported the presence of FMD in iliac arteries in eight patients and carotid FMD in two additional patients (60).

The high prevalence of FMD in SCAD patients clearly establishes a connection between the two conditions, primarily based on the understanding that the histopathological features of FMD weaken vessel walls, making them prone to dissection in the presence of precipitating factors. Indeed, the prevalence of FMD in non-atherosclerotic SCAD patients has been found to be over 70% (48).

The occurrence of extra-coronary vascular abnormalities in SCAD patients with FMD ranges from 10% to 86%, raising important questions regarding diagnostic algorithms following a SCAD event (2). In a case series study involving 173 SCAD patients, the research focused on analyzing the prevalence of aneurysms, dissections, and tortuosity in extracoronary arteries (61). The findings revealed that 32% of patients exhibited FMD, 8% had aneurysms, and 2% experienced dissections. Interestingly, there was a comparable prevalence of arterial tortuosity between the SCAD cases and the control group, and extracoronary vascular events were rare over a median 5-year follow-up period. Identifying such extra-coronary involvement and implementing appropriate preventive measures, especially for women planning pregnancy, is of utmost importance. Unfortunately, there is no clear consensus on the design of such measures, leaving clinicians to recommend the avoidance of known precipitating factors for SCAD, such as intense exercise, emotional stress, labor and delivery, intense Valsalva-type activities, drug abuse, and intense hormonal therapy in SCAD survivors.

### 4.4. SCAD in systemic autoimmune diseases

Systemic autoimmune diseases, especially systemic lupus erythematosus (SLE), have been suggested to potentially contribute to the pathogenesis of SCAD. The connection between inflammation and SCAD, as discussed in previous sections, remains a subject of debate. It's uncertain whether inflammation acts as a causal factor or is simply an expected response to an intimal tear or intramural hemorrhage. Randomized trials are lacking to definitively establish the relationship and associated risks between SCAD and systemic autoimmune diseases.

Saw et al. reported that within a cohort of 168 SCAD patients, 8.9% had concomitant systemic inflammatory conditions, including ulcerative colitis, Crohn's disease, rheumatoid arthritis, celiac disease, and Graves disease (48). A systematic literature review and meta-analysis by Ullah et al. identified only 10 cases of SCAD related to systemic autoimmune diseases, with 70% of these patients having SLE. These cases predominantly presented as non-ST-elevation myocardial infarctions (NSTEMI) and exhibited a higher-than-expected rate of percutaneous coronary intervention (PCI) with overall satisfactory outcomes (62). In a large Canadian cohort analysis of 750 SCAD patients, a 4.7% prevalence of systemic inflammatory conditions was observed (27).

However, in a case-control study involving 114 SCAD cases, systemic autoimmune diseases appeared to have a similar prevalence in SCAD patients compared to controls. This study did not reveal any significant relationship between systemic autoimmune diseases and SCAD, suggesting that SCAD may not have an inflammatory basis and that routine screening for systemic autoimmune diseases may be unnecessary (63).

It's important to note that the existing literature on the relationship between SCAD and systemic autoimmune disorders is limited and primarily comprises case reports and observational studies, making it difficult to draw definitive conclusions. Future prospective studies that investigate various systemic autoimmune disorders and explore autoantibody interactions with vessel wall structures as potential causes of SCAD will offer more insights into this possible pathophysiological pathway. Notably, a recent case-control study by Civieri et al. reported a significantly higher prevalence of autoantibodies targeting angiotensin-II receptor type 1 and endothelin-1 receptor type A in SCAD patients compared to controls (including healthy individuals and those with atherosclerotic CAD), emphasizing the potential role of autoimmunity in SCAD pathophysiology (64).

**Figure 3:**
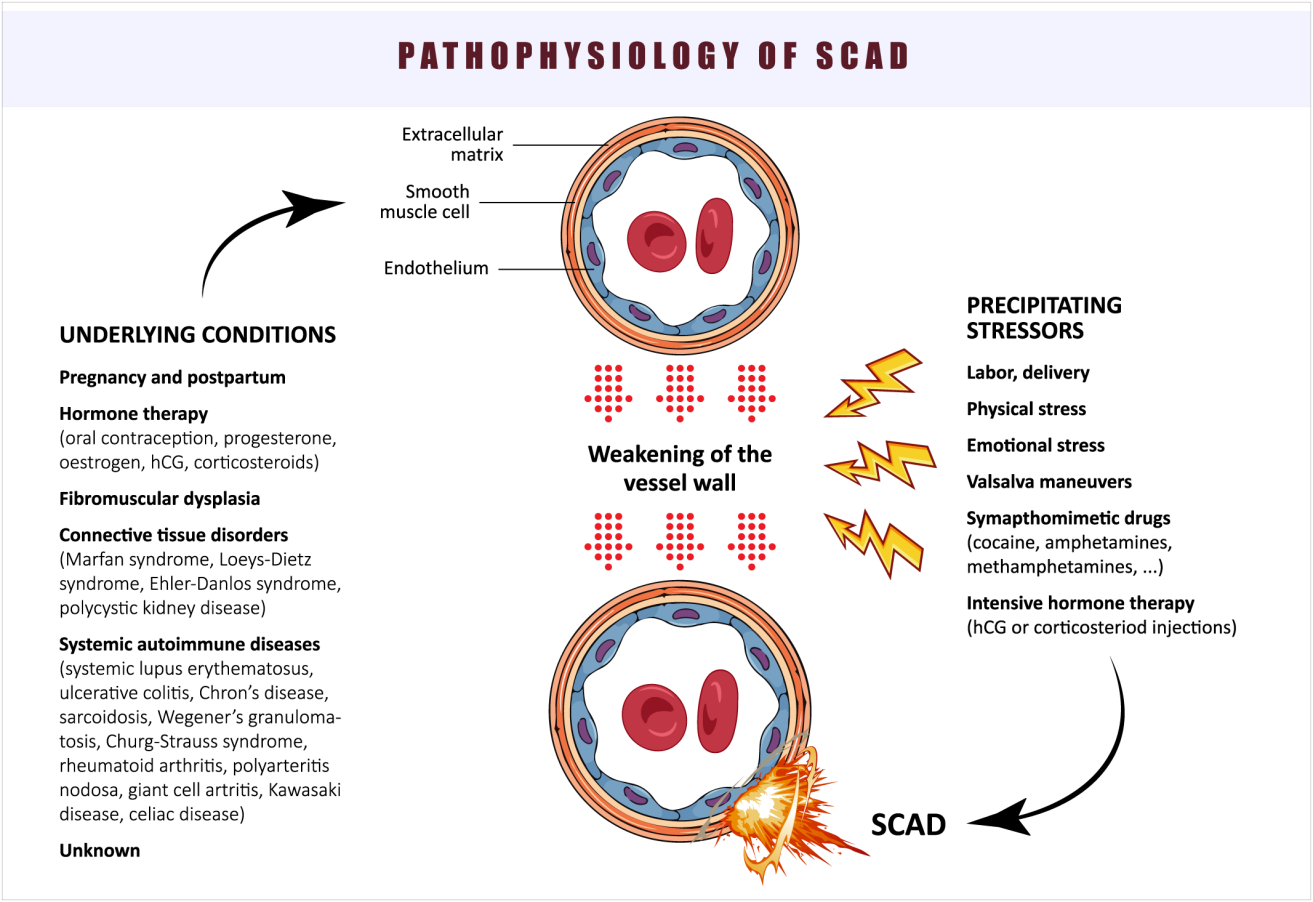
Pathophysiology of SCAD.

The proposed pathophysiology of SCAD is summarized in Figure 3.

## 5. Clinical presentation and diagnosis of SCAD

SCAD remains an underdiagnosed condition, and many patients may not present to medical services due to mild symptoms or SCD as the first manifestation. Among those who do present, a majority present with ACS, with 26%–55% presenting as ST-elevation myocardial infarction (STEMI) (48, 65). Typical chest pain is reported in 60%–90% of cases (66). However, some cases may be asymptomatic or have minimal symptoms, leading to delayed diagnosis or misinterpretation of angiographic findings (10).

Angiographic findings are crucial for the diagnosis of SCAD, and the classification proposed by Saw et al. is often used (1). However, there can be a misinterpretation of Type 3 SCAD as atherosclerotic CAD. Simultaneous multivessel dissection may occur in 10% of cases, emphasizing the need for careful evaluation of all coronary arteries. SCAD patients also have a higher prevalence of significant coronary artery tortuosity compared to the general population, which may be related to the presence of FMD and a higher risk of recurrence (67).

Intracoronary imaging techniques such as intravascular ultrasound (IVUS) and OCT have expanded our understanding of SCAD (68, 69). IVUS allows for the visualization of true and false lumens and the extension of intramural hematoma, while OCT provides high-resolution images of the vessel wall structure, location of intimal tear, and intramural hematoma size (70–72). These imaging modalities are particularly useful during percutaneous coronary intervention (PCI) to guide wire placement and stent sizing.

While non-invasive diagnostic tools for SCAD have their merits, their utility is somewhat limited. Echocardiography offers the ability to assess regional wall kinetics and left ventricular ejection fraction. On the other hand, cardiac magnetic resonance (CMR) imaging serves as a valuable tool for confirming SCAD or distinguishing it from other conditions, such as myocarditis or Takotsubo cardiomyopathy in type 4 SCAD (73, 74). However, it's important to note that normal CMR findings do not definitively rule out SCAD. CCTA, while less commonly employed in SCAD diagnosis, may find a role in follow-up due to its non-invasive nature and lower risk of catheter-induced injury. Nevertheless, interpreting CCTA findings can pose challenges, as intramural hematomas can sometimes be mistaken for artifacts, and non-calcified atherosclerotic plaques might be inaccurately interpreted as SCAD.

In summary, SCAD diagnosis relies on angiographic findings, with IVUS and OCT providing additional information on the vessel wall structure and intramural hematoma. Non-invasive imaging modalities such as echocardiography, CMR, and CCTA can aid in the assessment and follow-up of SCAD patients, but their diagnostic utility is limited compared to invasive techniques.

## 6. Management and outcomes of SCAD

The management of SCAD is primarily conservative, as natural healing evolution is observed in many cases (75–77). The majority of SCAD patients are treated conservatively, with no significant difference in MACE between patients with or without percutaneous coronary intervention (PCI) at the index hospitalization (65). Prolonged monitoring in the acute phase is recommended, as some studies have shown that not all conservatively treated patients experience complete healing (2, 76, 77).

The paucity of randomized trials focusing on medical therapy for SCAD has left treatment approaches primarily reliant on empirical strategies. The use of dual antiplatelet therapy (DAPT) in combination with anticoagulant therapy poses a potential risk of hematoma propagation. However, certain studies have documented the presence of intramural thrombus at the site of SCAD (71), providing a rationale for considering DAPT, preferably with a less potent P2Y12 inhibitor, for a duration of 12 months. Conversely, an analysis from the DISCO registry observed a higher incidence rate of adverse cardiovascular outcomes among 132 SCAD patients treated with DAPT (62.9% on aspirin plus clopidogrel, 36.4% on aspirin plus ticagrelor) compared to 67 SCAD patients managed with either aspirin 100 mg or a P2Y12 inhibitor (78). The authors of this study concluded that DAPT might pose a greater risk compared to single antiplatelet therapy for conservatively managed patients. This observation supports the hypothesis that more potent antiplatelet therapy could potentially induce intramural hematoma propagation.

While the use of aspirin therapy lacks a robust foundation in SCAD management, beta-blockers remain a cornerstone in medical treatment to mitigate the potential adverse effects of catecholamine surges. However, these therapeutic regimens are largely empirical and continue to evolve based on individualized approaches. The forthcoming results of the inaugural prospective, randomized, open-label, blinded-endpoint clinical trial, the Beta-blockers and Antiplatelet agents in patients with Spontaneous Coronary Artery Dissection (BA-SCAD), aimed at evaluating the efficacy of pharmacological therapy in SCAD patients, hold the promise of shedding light on this matter (79).

Patients presenting with left ventricular systolic dysfunction should receive treatment in accordance with the latest clinical guidelines. The use of statins in the context of SCAD remains a topic of debate, as some studies have indicated an elevated risk of recurrence, while others have reported lower recurrence rates among individuals using statins (60, 80).

The consideration of PCI in SCAD cases should be judicious, primarily for those experiencing prolonged symptoms and persistent ischemia. In a study conducted by Kotecha et al., a comparative analysis involving 215 SCAD patients who underwent PCI and a matched cohort of SCAD patients managed conservatively was performed (81). The study findings indicated that high-risk SCAD cases [excluding those presenting with STEMI/cardiac arrest, thrombolysis in myocardial infarction (TIMI) 0/1 flow, or proximal dissection] received stents of greater length and exhibited a higher propensity for PCI-related complications. However, it was notable that this subgroup demonstrated more pronounced improvements in coronary flow and exhibited favorable medium-term outcomes concerning major adverse cardiovascular and cerebrovascular events. This assertion is corroborated by the most recent recommendations provided in the guidelines for the management of ACS by the European Society of Cardiology. According to these guidelines, PCI in SCAD is advised solely for patients exhibiting symptomatic manifestations and indicators of ongoing ischemia, coupled with a substantial extent of myocardium at risk and diminished anterograde flow. This recommendation is categorized as Class I, supported by Level of Evidence C (82) Careful techniques, such as balloon angioplasty without stent deployment or the use of specific stent techniques to avoid hematoma propagation, may be employed (53, 83). Surgical revascularization with coronary artery bypass grafting is performed in specific cases, such as PCI failure, left main involvement, or multivessel presentation.

The main concern in the follow-up of SCAD patients is the risk of recurrence. Close monitoring and avoidance of known precipitating factors, such as intense exercise and emotional stress, are recommended. In the case of pregnancy-related SCAD, future pregnancies should be carefully monitored, and non-hormonal contraception methods are recommended. Regular, moderate exercise is proposed for the overall well-being of SCAD patients, despite physical activity being identified as a risk factor for SCAD development.

Overall, further research is needed to better understand the healing process, identify underlying diseases such as fibromuscular dysplasia and connective tissue disorders, and establish optimal management and follow-up protocols for SCAD patients.

## 7. Future directions and research needs

Future research on SCAD should focus on several key areas to advance our understanding and improve patient outcomes:

1. Pathophysiology and Etiology: Further studies are needed to elucidate the underlying mechanisms and causes of SCAD. This includes investigating the role of hormonal factors, inflammation, genetic predisposition, connective tissue disorders, and autoimmune diseases in the development of SCAD. Identifying specific genetic mutations and molecular pathways associated with SCAD will help in early diagnosis, risk stratification, and targeted therapies.
2. Diagnostic Tools and Algorithms: Developing clear diagnostic algorithms and guidelines for the accurate and timely diagnosis of SCAD is crucial. This involves refining the use of angiography, intravascular imaging techniques (such as IVUS and OCT), non-invasive imaging modalities (such as CMR and CCTA), and genetic testing to differentiate SCAD from other conditions and identify underlying diseases. Standardized criteria for the interpretation of imaging findings and histopathological features of SCAD are needed.
3. Medical Management: Conducting randomized controlled trials to evaluate the efficacy and safety of different medical treatments for SCAD is essential. This includes investigating the optimal duration of DAPT, the role of anticoagulants, the use of beta-blockers and other medications, and the potential benefits of statins in preventing recurrence. Long-term follow-up studies are necessary to assess the outcomes and recurrence rates associated with different treatment strategies.
4. Risk Stratification and Prevention: Developing risk stratification tools to identify patients at higher risk of SCAD recurrence, adverse cardiovascular events, or complications is crucial for personalized management. Understanding the role of lifestyle factors, such as exercise intensity and emotional stress, in triggering SCAD and implementing preventive measures to avoid these triggers is important. Identifying modifiable risk factors and implementing targeted preventive strategies can help reduce the burden of SCAD.
5. Pregnancy and Women's Health: Further research is needed to understand the specific risks and management strategies for pregnancy-related SCAD. This includes investigating the impact of hormonal changes, vascular adaptations during pregnancy, and the use of hormonal therapies and contraceptives on SCAD risk. Developing guidelines and recommendations for family planning and pregnancy management in women with a history of SCAD is necessary.
6. Patient Education and Support: Improving patient awareness, education, and support systems for SCAD are crucial. Providing information on symptoms, risk factors, and preventive measures can help patients recognize early signs and seek appropriate medical attention. Creating patient support networks and resources can enhance patient well-being and improve long-term outcomes.
7. Collaborative efforts between researchers, clinicians, and patient advocacy groups are essential to address these research needs and advance our understanding of SCAD. By conducting high-quality studies and sharing data through registries and international collaborations, we can make significant progress in the diagnosis, management, and prevention of SCAD.

## 8. Conclusion

In conclusion, SCAD is a unique and complex condition that predominantly affects women, often in their reproductive years. It is characterized by the formation of intramural hematoma, with or without intimal tear, and potential obstruction of blood flow. The recognition and understanding of SCAD have increased in recent years, but many aspects of its pathophysiology, diagnosis, and management remain uncertain.

SCAD is associated with various underlying conditions such as FMD, CTDs, systemic autoimmune diseases, and hormonal changes during pregnancy. Further research is needed to explore the exact mechanisms by which these factors contribute to SCAD development and to develop specific diagnostic algorithms for early and accurate diagnosis.

Management of SCAD primarily involves conservative measures, with a focus on close monitoring and supportive care. While some cases may require invasive interventions such as PCI or coronary artery bypass grafting, the majority of SCAD patients can be managed conservatively. Research is needed to determine the optimal medical therapies and preventive strategies to reduce the risk of recurrence and improve long-term outcomes.

Collaborative efforts between researchers, clinicians, and patient advocacy groups are essential to advance our understanding of SCAD and namely elucidate the exact etiology. Future studies should focus on clarifying the underlying mechanisms, improving diagnostic tools, developing evidence-based treatment approaches, and identifying effective strategies for risk stratification and prevention. By addressing these research needs, we can enhance the management and outcomes of SCAD patients and provide them with appropriate support and care.

## 9. Limitations

The primary limitation of our review paper is that we did not conduct a systematic review of the literature. Instead, our review took a more focused approach, aiming to synthesize and discuss key concepts and findings within a narrower scope. This might have led to potential omissions of relevant studies that a systematic review would have captured. Furthermore, the absence of a systematic review methodology might impact the overall rigor and comprehensiveness of our review. By not adhering to this methodology, our review paper could be susceptible to selection bias and may not provide a complete and unbiased overview of the literature on the topic. In light of these limitations, we acknowledge that the conclusions drawn in our review should be interpreted with caution. While we aimed to provide valuable insights and perspectives within the chosen scope, the absence of a systematic approach may limit the generalizability and robustness of our findings.
